# Impact of p120-catenin Isoforms 1A and 3A on Epithelial Mesenchymal Transition of Lung Cancer Cells Expressing E-cadherin in Different Subcellular Locations

**DOI:** 10.1371/journal.pone.0088064

**Published:** 2014-02-04

**Authors:** Yijun Zhang, Yue Zhao, Guiyang Jiang, Xiupeng Zhang, Huanyu Zhao, Junhua Wu, Ke Xu, Enhua Wang

**Affiliations:** 1 Department of Pathology, First Affiliated Hospital and College of Basic Medical Sciences, China Medical University, Shenyang, China; 2 Department of Radiology, First Affiliated Hospital of China Medical University, Shenyang, China; Aix-Marseille University, France

## Abstract

The epithelial mesenchymal transition (EMT) is an important process in tumor development. Despite previous investigations, it remains unclear how p120-catenin (p120ctn) isoforms 1A and 3A affect the EMT of tumor cells. Here we investigated expression of p120ctn, E-cadherin and vimentin in 78 human non-small cell lung cancer (NSCLC) samples by immunohistochemistry and found that p120ctn membrane expression positively correlated with E-cadherin expression (*P*<0.001) and negatively correlated with vimentin expression and lymph node metastasis (*P*<0.05). Meanwhile, p120ctn cytoplasmic expression negatively correlated with E-cadherin expression (*P*<0.001) and positively correlated with vimentin expression and lymph node metastasis (*P*<0.05). Cells expressing high (H460 and SPC) and low (H1299 and LK2) levels of p120ctn were screen to investigate its impact on EMT. E-cadherin was restricted to the cell membrane in H460 and H1299 cells, whereas it was expressed in the cytoplasm of SPC and LK2 cells. Ablation of endogenous p120ctn isoform 1A in cells expressing high levels of the protein resulted in decreased E-cadherin expression, increased N-cadherin, vimentin and snail expression and enhanced invasiveness in H460 cells. Meanwhile, completely opposite results were observed in SPC cells. Furthermore, transfection of in H1299 cells expressing low p120ctn levels with the p120ctn isoform 1A plasmid resulted in increased E-cadherin expression, decreased N-cadherin, vimentin and snail expression and weakened invasiveness, while LK2 cells showed completely opposite results. Both cell lines expressing low p120ctn levels and transfected with the p120ctn isoform 3A plasmid appeared to have increased E-cadherin expression, decreased N-cadherin, vimentin and snail expression and weakened invasiveness. In conclusion, in cells with membrane E-cadherin, both p120ctn isoforms 1A and 3A inhibited EMT and decreased cell invasiveness. In cells with cytoplasmic E-cadherin, p120ctn isoform 1A promoted EMT and increased cell invasiveness, while p120ctn isoform 3A inhibited the EMT and decreased cell invasiveness.

## Introduction

The epithelial mesenchymal transition (EMT) is a rapid and often reversible change of cell phenotype and plays a particularly important role in tumor development. In the process of EMT, epithelial cells undergo a phenotypic switch to form mesenchymal cells that are similar in appearance to fibroblasts [1], [2]. Such phenotypic changes cause epithelial cells to lose their characteristic cell-cell adhesion structures, alter their polarity, modulate the organization of their cytoskeletal systems, switch expression from keratin- to vimentin-type intermediate filaments, as well as become isolated, motile and resistant to anoikis [3], [4]. Typically, cells undergoing EMT show decreased E-cadherin expression [5], [6] and decreased expression of mesenchymal biomarkers, such as N-cadherin, vimentin, snail, slug and twist [7], [8].

Previous studies on the relationship between p120-catenin (p120ctn) and EMT have been confined to the switch from short to long p120ctn isoforms during the EMT induced by expression of SIP1/ZEB2 [9], twist [10] or Zeppo1 [11]. However, the mechanism by which p120-catenin isoforms 1A and 3A affect EMT of tumor cells remains unknown. The p120ctn protein has four isoforms (1 to 4) resulting from four transcriptional start sites, and each isoform has a full central Armadillo repeat domain that can interact with the juxtamembrane domain of E-cadherin in order to participate in the formation of an adhesion complex on the cell membrane [12]. These observations suggest that the subcellular localization and function of p120ctn can be affected by the localization of E-cadherin. Previous studies have shown that p120ctn may play opposing roles depending on whether it is located on the membrane or in the cytoplasm of cells [13], [14]. Others have also found that p120ctn isoforms 1A and 3A have different regulatory functions on tumor cell proliferation, invasion and metastasis [15], [16], . These studies indicate that if p120ctn has an impact on the EMT, it is likely to be different between p120ctn isoforms 1A and 3A.

Some studies have shown that p120ctn may promote or inhibit tumor growth and invasiveness depending on whether E-cadherin expressed or not [18], [19]. Yu and colleagues also found different effects of p120ctn isoforms 1A and 3A on proliferation and invasion in tumor cells exhibiting different localizations of E-cadherin [20]. Thus, whether p120ctn isoforms 1A and 3A also play different roles in regulating EMT in tumor cells with E-cadherin at different locations remains unknown.

The aim of this study was to determine the potential effects and regulatory mechanisms of p120ctn isoforms 1A and 3A on EMT in lung cancer cells. We first revealed that the membrane or cytoplasmic expression of p120ctn correlated with expression of E-cadherin and vimentin or lymph node metastasis by immunohistochemistry. We further detected the expression levels of p120ctn, E-cadherin and vimentin in lung cancer cells by Western blot and screened cell lines expressing both low and high levels of p120ctn and with E-cadherin in the membrane or cytoplasm. Changes in expression of EMT-related molecules and cell invasion were also investigated by knockdown of endogenous p120ctn-1A or overexpression by transfection of p120ctn-1A and 3A plasmids into cells.

## Materials and Methods

### Materials

This study was conducted with the approval of the institutional review board at China Medical University. Written consent was given by the participants for their information to be stored in the hospital database and for their specimens to be used in this study. All clinical investigations were conducted according to the principles expressed in the Declaration of Helsinki. Samples were collected from 78 cases of squamous cell lung cancer and lung adenocarcinoma diagnosed at the First Affiliated Hospital of China Medical University (Sheny-ang, China). The samples were from 46 male and 32 female patients with an average age of 57 years. The samples were classified according to lung tumor histological criteria (2004) of the World Health Organization (WHO) [21] as squamous cell lung carcinoma (32 cases) or lung adeno-carcinoma (46 cases). Thirty cases were highly differentiated, and forty-eight were moderately or poorly differentiated. Lymph node metastases were present in 43 cases, but not in the other 35. We selected cases with lymph node metastases to compare the metastatic nodules with the primary tumor. Tumor staging was performed according to the tumor-node-metastasis (TNM) staging system of the International Union against Cancer (UICC) [22]. There were 39 cases at stage I–II, and 39 cases at stage IIIa–IIIb. None of the patients had received radiotherapy or chemotherapy before the operation and were given the standard treatment following the surgery. All samples were fixed in formalin, embedded in paraffin and stained with hematoxylin and eosin for pathological analysis and diagnosis.

### Cell culture

Normal human bronchial epithelial (HBE) cells and A549, H1299, H460 and H157 cell lines were obtained from the American Type Culture Collection (Manassas, VA, USA). The SPC-A-1, LTEP-A-2 and LK2 cell lines were purchased from the Shanghai Cell Bank of Chinese Academy of Science. The human lung ADC Anip973 and AGZY83a cell lines were purchased from Shanghai Bioleaf Biotech Co., Ltd (http://www.bioleaf.com) and stored in the Department of Pathology, Harbin Medical University. Cells were cultured in RPMI 1640 (Invitrogen, Carlsbad, CA, USA) containing 10% fetal calf serum (Invitrogen), 100 IU/ml penicillin (Sigma, St. Louis, MO, USA) and 100 mg/ml streptomycin (Sigma).

### Plasmid construction and transfection

Expression plasmids for p120ctn isoforms 1A and 3A (donated by Dr. Albert B. Reynolds Department of Cancer Biology, Vanderbilt University School of Medicine, TN, USA) have been described previously [16]. Sequences of p120ctn-1A-siRNA (Guangzhou Ruibo Co. Ltd, Guangzhou, China) used in the experiments were as follows: si-h-CTNND1: 5′-CACAAGAUGCCAACCCACU dTdT-3′, 3′-dTdT GUGUUCUACGGUUGGGUGA-5′. The cells were transiently transfected with p120ctn-1A-siRNA and plasmids expressing p120ctn isoforms 1A and 3A using Lipofectamine 2000 (Invitrogen, Carlsbad, CA) or Attractene Transfection Reagent (QIAGEN GmbH, Hilden, Germany) according to the manufacturer's instructions.

### Immunohistochemistry

The paraffin embedded samples were cut serially into 4-μm thick sections. Normal bronchial epithelium present in the tumor slides was used as an internal positive control. Immunostaining was performed by the streptavidin-peroxidase (S-P) method. The tissue sections were incubated with a p120ctn mouse monoclonal antibody (1∶100, cat. 610134, BD Transduction Laboratories, Lexington, KY, USA), E-cadherin rabbit monoclonal antibody (1∶100, cat. SC-7870; Santa Cruz Biotechnology, Santa Cruz, CA, USA) or vimentin rabbit monoclonal antibody (ready-to-use, cat. RMA-0547, MaiXin Bio, Fuzhou, China) at 4°C overnight. PBS was used as a negative control. Biotinylated goat anti-mouse serum IgG or biotinylated goat anti-rabbit serum IgG (ready-to-use, cat. KIT-9922, MaiXin Bio) was used as the secondary antibody. After washing, the sections were incubated with streptavidin–biotin conjugated with horseradish peroxidase (Ultrasensitive, MaiXin Bio), and then the peroxidase reaction was developed with 3,3-diaminobenzidine tetrahydrochloride (MaiXin Bio). Light counterstaining was performed with hematoxylin, and then the sections were dehydrated in alcohol before being mounted.

Two investigators independently examined all the tumor slides. Five random fields were examined per slide, and 100 cells were observed per high magnification field (400×). The percentage of positive cells was scored as follows: 0 = no staining; 1+ = 0–25%; 2+ = 26–50%; 3+ = 51–75%; and 4+ = 76–100%. The staining intensity was scored as follows: 0 = no staining; 1 = light yellow granules; 2 = dark yellow or brown granules. The labeling score defined by multiplying the percentage of positive cells by the staining intensity was the final score for the section. When the total score was ≥3, the case was defined as positive. When the total score was <3, the case was defined as negative. For scores greater than 3 points, when more than 30% of the tumor cells stained strongly and continuously for p120ctn signal on the cell membrane, the sample was defined as membrane positive. When fewer than 30% of the tumor cells displayed membrane expression but stained strongly and continuously for p120ctn in the cytoplasm, the sample was defined as cytoplasm positive.

### Western blot analysis

Fifty micrograms of proteins were separated by SDS-PAGE (10%). After transfer to a polyvinylidene fluoride (PVDF) membrane (Millipore, Billerica, MA, USA), the proteins were incubated overnight at 4°C with antibodies to the following: p120ctn (1∶500, cat. 610134), E-cadherin (1∶300, cat. 610181), N-cadherin (1∶1000, cat. 610920) (BD Transduction Laboratories, Lexington, KY, USA), vimentin (1∶1000, cat. 5741), snail (1∶500, cat. 3879) (Cell Signaling Technology, Boston, MA, USA) and twist (1∶200, cat. sc-15193, Santa Cruz Biotechnology). After incubation with anti-mouse (1∶2000, E030110-01) or anti-rabbit (1∶2000, E030120-01) IgG (EarthOx LLC, San Francisco, CA, USA) at 37°C for 2 h, the protein bands were visualized using enhanced chemiluminescence (ECL, Thermo Fisher Scientific, Waltham, MA, USA) and quantified using BioImaging Systems (UVP, Upland, CA, USA). Relative protein levels were calculated in reference to GAPDH as the loading control.

### Immunofluorescent staining

Cells grown on glass coverslips were fixed with ice-cold 4% paraformaldehyde for 15 min, followed by permeabilization with 0.2% Triton X-100 and incubation with normal goat serum for 30 min at 37°C. Cells were then incubated overnight with p120ctn mouse monoclonal antibody (1∶200, cat. 610134; BD Transduction Laboratories, Lexington, KY, USA) and E-cadherin rabbit polyclonal antibody (1∶100, SC-7870; Santa Cruz Biotechnology). Primary antibodies were applied overnight at 4°C, followed by incubation with a rhodamine/fluorescein-5-isothiocyanate (FITC)-labeled secondary antibody goat anti-mouse or TRITC-labeled goat anti-rabbit IgG (1∶100, cat. E031210-01 and E031320-01, EarthOx, San Francisco, CA, USA). The nuclei were counterstained with propidium iodide/4, 6 diamidino-2-phenylin-dole. Epifluorescent microscopy was performed using an inverted Nikon TE300 microscope (Melville, NY, USA), and confocal microscopy was performed using a Radiance 2000 laser scanning confocal microscope (Carl Zeiss, Thornwood, NY, USA).

### Matrigel cell invasion assay

Matrigel cell invasion assays were performed according to the manufacturer's instructions (Corning, Acton, MA, USA). A 100-μl cell suspension (5×10^5^ cells) was added to the upper chamber, while the lower chamber was filled with RPMI 1640 medium containing 10% fetal calf serum. Each upper and lower chamber was separated by a 8-μm porous polycarbonate membrane. The cells were incubated for 24 h at 37°C in a humid atmosphere with 5% CO_2_. After the medium was discarded, the cells were fixed with methanol for 30 min and stained with hematoxylin (Sigma). For each filter, the numbers of cells that invaded to the lower surface of the porous membrane in five different fields of 400× magnification were counted randomly using a Nikon E200 microscope. The mean was calculated from data obtained from each experiment repeated three times.

### Statistical analysis

All statistical analyses were performed using SPSS 17.0 (SPSS Inc., Chicago, IL, USA) for Windows software. The chi-square test was used to analyze immunohistochemistry data. The independent samples T test was used to examine transwell experimental data. *P* values<0.05 were considered statistically significant.

## Results

### Membrane expression of p120ctn positively correlates with E-cadherin expression and negatively correlates with vimentin expression and lymph node metastasis

**Figure 1:**
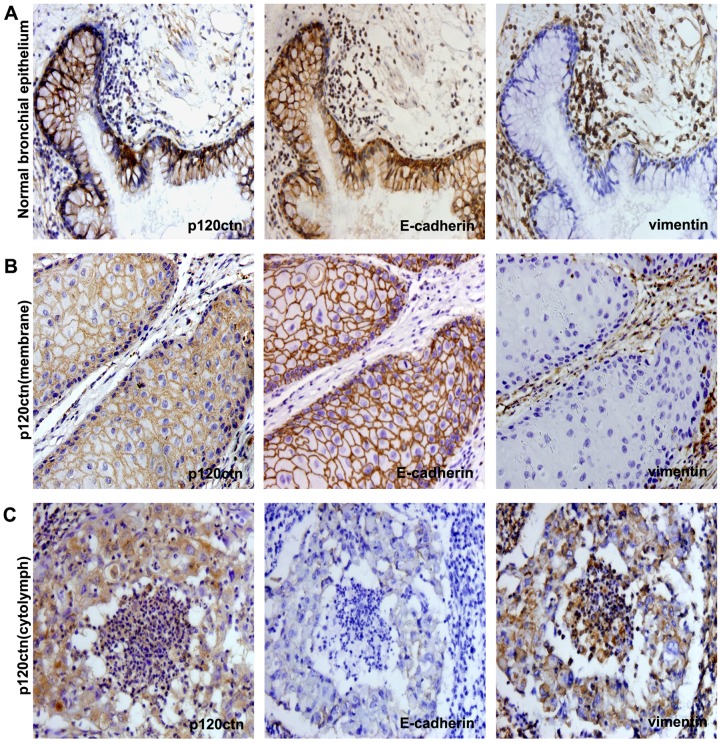
Immunohistochemical analysis of p120ctn, E-cadherin and vimentin localization in NSCLC. (A) E-cadherin and p120ctn were membrane positive, and vimentin was negative in normal bronchial epithelial cells. **(B)** E-cadherin was membrane positive, and vimentin was negative in p120ctn membrane-positive lung cancer cells. **(C)** E-cadherin was negative, and vimentin was positive in p120ctn cytoplasmic-positive lung cancer cells.

Normal bronchial epithelial tissues showed p120ctn in the membrane (Figure 1A), while the proportion of lung cancer tissues expressing p120ctn in the membrane was significantly lower (35%, 27/78) than that with p120ctn cytoplasmic expression (65%, 51/78). E-cadherin was expressed in the membrane in normal bronchial epithelium tissues (Figure 1A), while the rate of positive expression was decreased (28%, 22/78) and that of negative expression was significantly increased (72%, 56/78) for E-cadherin in lung cancer tissues. Vimentin was negatively expressed in normal bronchial epithelial tissues (Figure 1A), while the rate of positive expression was increased to 32% (25/78) in the lung cancer tissue. It appears lung cancer tissues with cytoplasmic/nuclear localization of p120ctn tended to express vimentin in comparison with those with the membranous localization (41.2% [21/51] versus 14.8 [4/27]).. Cytoplasmic/nuclear localization of p120ctn showed increased lymph node metastasis (29/51) in comparison with the membranous localization (8/27). Statistical analysis showed that the localization of p120ctn was closely related with E-cadherin expression, vimentin expression and lymph node metastasis (*P*<0.05) (Table 1). In other words, p120ctn membrane expression was positively correlated with E-cadherin expression and negatively correlated with vimentin expression and lymph node metastasis (Figure 1B); meanwhile, p120ctn cytoplasmic expression was negatively correlated with E-cadherin expression and positively correlated with vimentin expression and lymph node metastasis (Figure 1C).

**Table 1:** Correlation between E-cadherin, vimentin and lymph node metastasis and p120ctn.

		p120ctn			
	N	membrane	cytolymph/nucleolus	X2	p
E-cadherin					
negative	56	9	47	30.166	<0.01
positive	22	18	4		
Vimentin					
negative	53	23	30	5.633	0.022
positive	25	4	21		
Lymph node metastasis					
No	41	19	22	5.251	0.032
Yes	37	8	29		

### Localization of p120ctn is consistent with E-cadherin in lung cancer cells

**Figure 2:**
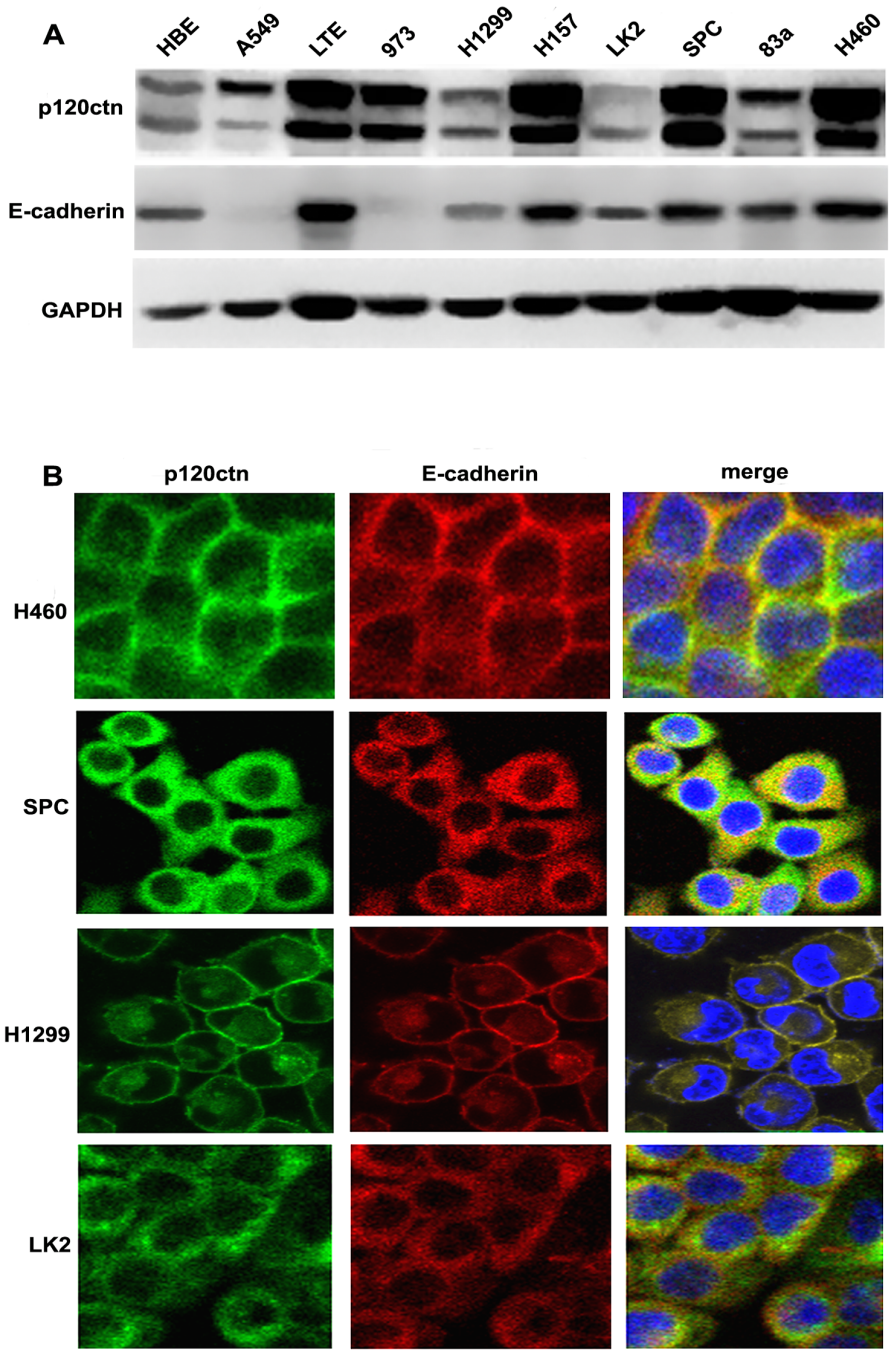
Expression and localization of p120ctn and E-cadherin in H460, SPC, H1299 and LK2 cells. (A) Western blot analyses showed expression of p120ctn and E-cadherin in nine lung cancer cell lines and HBE. **(B)** By immunofluorescence analysis, the expression of E-cadherin and p120ctn were observed restricted to the cell membrane at cell-cell adherens junctions in H460 and H1299 cells, whereas they both were confined to the cytoplasm in SPC and LK2 cells.

We examined the protein expression levels of p120ctn and E-cadherin in normal HBE cells and nine lung cancer cell lines by Western blot and found that they all expressed mainly isoforms 1A (120 kDa) and 3A (100 kDa) of p120ctn (Figure 2A). Although the protein expression levels of p120ctn were not related to E-cadherin, the localization (membrane or cytoplasm) of p120ctn was always consistent with that of E-cadherin. We then screened cells expressing high levels of p120ctn and E-cadherin in the membrane (H460 cells) or cytoplasm (SPC cells), as well as those expressing low levels of p120ctn and E-cadherin in the membrane (H4299 cells) or cytoplasm (LK2 cells) for further study (Figure 2B).

### Different functions of p120ctn isoform 1A in EMT are dependent on E-cadherin subcellular localization

**Figure 3:**
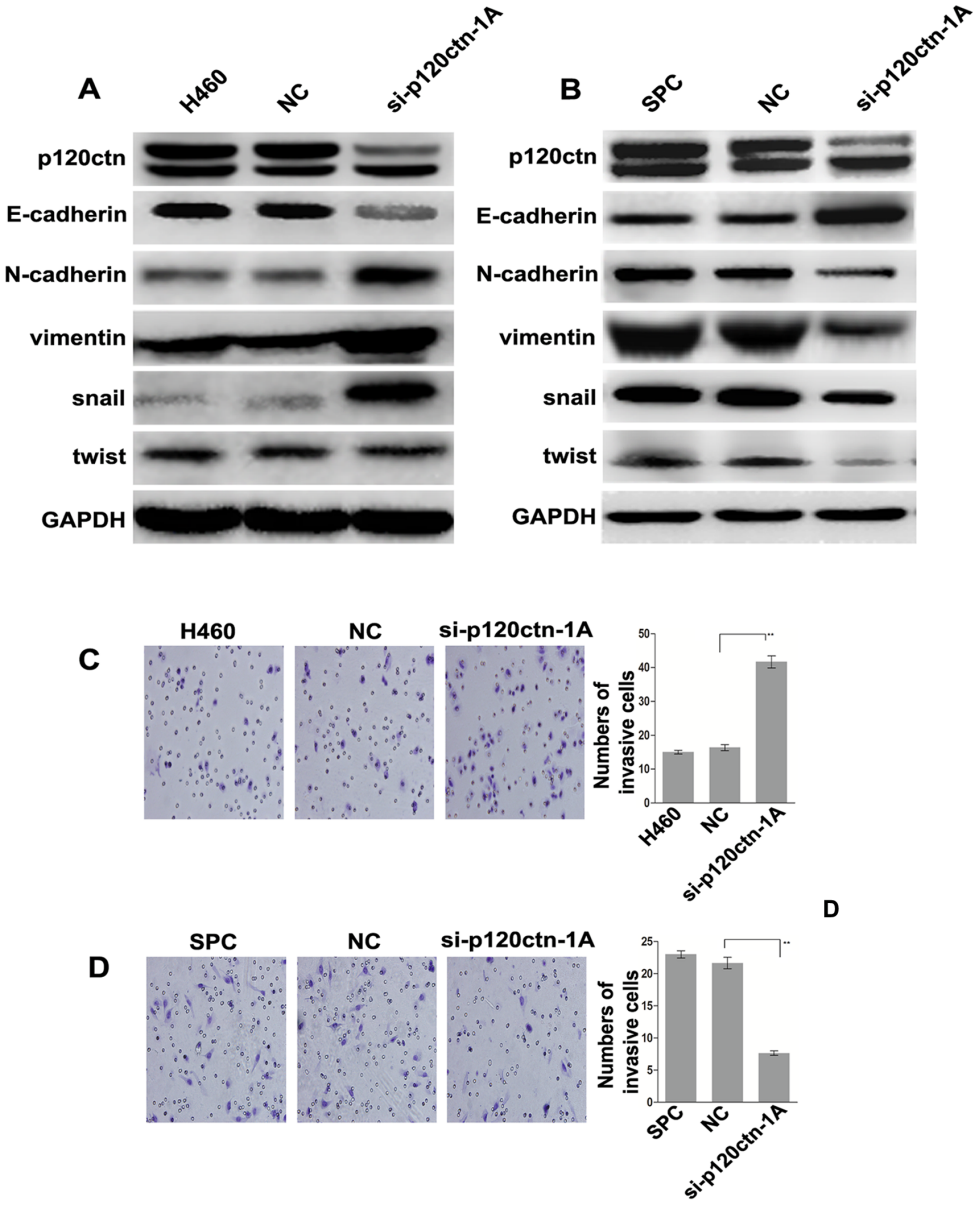
p120ctn isoform 1A plays a different role in regulating EMT in H460 and SPC cells. (A) Ablation of p120ctn isoform 1A decreased E-cadherin expression and increased N-cadherin, snail and vimentin expression in H460 cells. (B) SPC cells were treated as in (A) and the opposite results were obtained. (C) Ablation of p120ctn isoform 1A enhanced the invasiveness of H460 cells (**P<0.01). (D) Ablation of p120ctn isoform 1A decreased the invasiveness of SPC cells (**P<0.01).

Knockdown of endogenous p120ctn isoform 1A by siRNA-p120ctn-1A resulted in decreased E-cadherin expression and increased N-cadherin, snail and vimentin expression in H460 cells (Figure 3A). However, knockdown of endogenous p120ctn-1A by siRNA-p120ctn-1A showed opposite results in SPC cells, where we found increased E-cadherin expression and decreased N-cadherin, snail and vimentin expression (Figure 3B). In comparison with the control, the ablation of p120ctn isoform 1A also enhanced the H460 cells invasiveness (17.33±1.25 vs. 36.33±1.70, *P*<0.01) (Figure 3C), whereas reduced the SPC cells invasiveness (23.0±0.82 vs. 13.0±0.82, *P*<0.01) (Figure 3D). These results revealed that the p120ctn isoform 1A plays a different role in EMT and cell invasiveness in different E-cadherin subcellular locations.

### Inhibitory function of p120ctn isoform 3A on EMT is not affected by differences in E-cadherin subcellular localization

**Figure 4:**
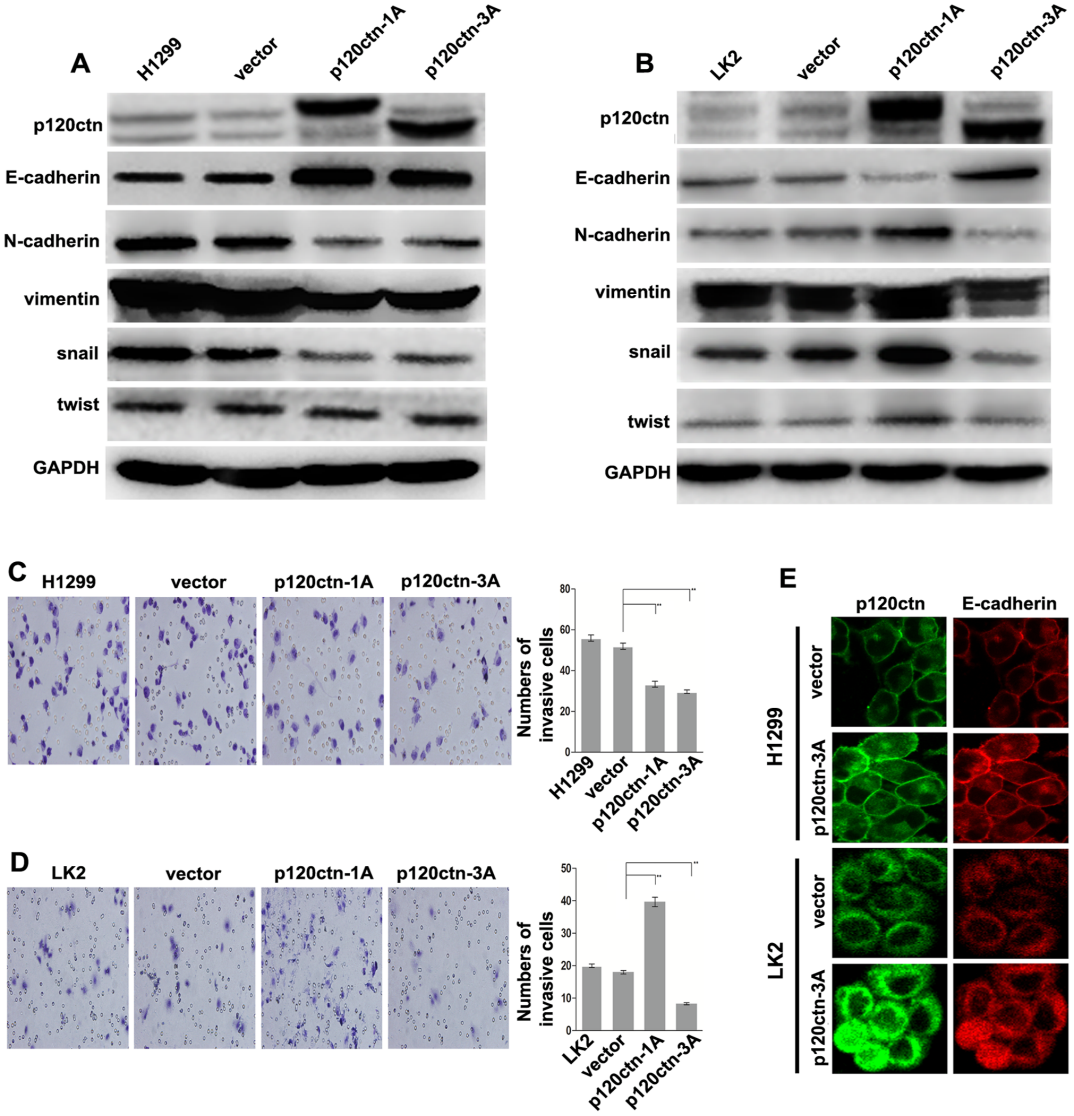
p120ctn isoform 3A maintains the role of inhibitiing EMT independently of E-cadherin localization. (A, B) Both H1299 (E-cadherin membrane localization) and LK2 cells (E-cadherin cytoplasmic localization) transiently transfected with the p120ctn isoform 3A plasmid showed increased E-cadherin expression and decreased N-cadherin, vimentin and snail expression. (C, D) Transient transfection of p120ctn isoform 3A plasmids into H1299 and LK2 cells resulted in decreased cell invasiveness (**P<0.01). (E) E-cadherin remained localized on the membrane in H1299 cells and in the cytoplasm of LK2 cells after transfection of the p120ctn isoform 3A plasmid.

To verify whether p120ctn isoforms 1A and 3A play different roles in regulating EMT, their expression plasmids were transiently transfected into lung cancer cells with low expression of p120ctn (H1299 with membrane E-cadherin expression and LK2 with cytoplasmic E-cadherin expression). The western-blot analysis demonstrated that overexpression of the p120ctn isoform 1A led to increased E-cadherin expression and decreased N-cadherin, vimentin and snail expression (Figure 4A); on the contrary, the decreased E-cadherin expression and increased N-cadherin, vimentin and snail expression were observed in LK2 cells (Figure 4B). Overexpression of the p120ctn isoform 1A also reduced the H1299 cell invasiveness (52.0±2.65 vs. 33.33±2.64, *P*<0.01) (Figure 4C), while enhanced the LK2 cell invasiveness (18.0±0.82 vs. 39.66±2.05, *P*<0.01) (Figure 4D).. Overexpression of p120ctn isoform 3A led to increased E-cadherin expression, decreased N-cadherin, vimentin and snail expression (Figure 4A, 4B) and reduced cell invasiveness (52.0±2.65 vs. 29.66±1.53, *P*<0.01; 18.0±0.82 vs. 8.33±expression 0.47, *P*<0.01) (Figure 4C, 4D) in both of these cell lines. These results further confirmed that the p120ctn isoform 1A had a different effect on EMT depending on the subcellular localization of E-cadherin. They also revealed that the p120ctn isoform 3A maintained an inhibitory role in the EMT of lung cancer cells whether E-cadherin was localized to the membrane or the cytoplasm.

## Discussion

The phenomenon of EMT in tumor cells often leads to decreased cell adhesion and increased mobility, and this transition is accompanied by decreased E-cadherin expression and increased expression of N-cadherin, vimentin and other mesenchymal biomarkers [3], [4], [5], [6], [7]. As an important factor for stabilizing E-cadherin, p120ctn plays a role in inhibiting or promoting tumor cell proliferation and invasion that is dependent on whether E-cadherin is expressed or not [16], [17]. Furthermore, p120ctn isoforms 1A and 3A have shown different effects on E-cadherin expression and tumor cell invasiveness which are based on differences in the localization of E-cadherin [18]. These results strongly suggest that p120ctn most likely regulates the EMT of tumor cells by affecting E-cadherin expression and that p120ctn isoforms 1A and 3A play different roles in EMT expressing E-cadherin in different subcellular locations.

We first found that the p120ctn membrane expression was positively correlated with E-cadherin expression and negatively correlated with vimentin expression and lymph node metastasis, while the cytoplasmic expression of p120ctn was negatively correlated with E-cadherin expression and positively correlated with vimentin expression and lymph node metastasis by immunohistochemistry. Although these results were consistent with previous studies [13], [14], they further suggested that p120ctn likely affects the EMT by influencing the expression of E-cadherin and vimentin and thereby the cell invasion and metastasis in non-small cell lung cancer (NSCLC).

To confirm the different impacts of p120ctn isoforms 1A and 3A on EMT in cells expressing E-cadherin in different locations, we selected H460 and H1299 cells with E-cadherin membrane expression and SPC and LK2 cells with E-cadherin cytoplasmic expression for further analysis. Plasmids expressing the p120ctn isoforms 1A and 3A were constructed, and the full-length p120ctn siRNA was synthesized for these experiments. Since the sequence beyond amino acids 1–101 of p120ctn isoform 1A is similar to that of p120ctn isoform 3A [24], [25], we could not design an interference sequence specifically for p120ctn isoform 3A. Therefore, we had to further study the impact of the two isoforms on EMT and cell invasiveness in lung cancer cells with different E-cadherin locations specifically by knocking down p120ctn isoform 1A in H460 and SPC cells with high p120ctn expression and transfecting cDNA plasmids for exogenous p120ctn isoforms 1A and 3A into H1299 and LK2 cells with low expression of p120ctn.

Knockdown of p120ctn isoform 1A in H460 cells destroyed the epithelial cell adhesion complexes. E-cadherin expression was also downregulated due to the loss of its important stabilizing factor, p120ctn isoform 1A, which was consistent with previous studies [20], [26]. Decreased E-cadherin expression and disrupted cell-cell adhesion may induce EMT [27], [28], [43], [44], which results in increased N-cadherin, vimentin and snail expression and enhanced cell invasiveness. On the other hand, overexpressed p120ctn isoforms 1A and 3A was shown to bind E-cadherin located on the membrane proactively in tumor cells [29] and then inhibit the degradation of E-cadherin and stabilize its expression, contributing to the formation of effective epithelial cell adhesion complexes [30], [31], [32]. As these series of processes maintained the normal cell-cell adhesion connection and inhibited EMT, there was increased E-cadherin expression and decreased N-cadherin, vimentin and snail expression, as well as inhibited cell invasiveness in H1299 cells.

Previous studies have shown that although p120ctn isoform 1A could bind E-cadherin in the cytoplasm, they could not form effective adhesion complexes on the membrane between epithelial cells [33]. Furthermore, the cytoplasmic E-cadherin is likely not to be the full-length E-cadherin but instead cleaved E-cadherin fragments, such as E-cad/sE-cad (80 kDa) and E-cad/CTF2 (33 kDa) [34]. The E-cad/CTF2 fragment can bind to p120 in the cytoplasm and then translocate into the nucleus and bind the transcriptional repressor of Kaiso to activate the Wnt/b-catenin pathway [35], [36], finally promoting the EMT of tumor cells and enhancing cell invasion and metastasis [37]. Moreover, others have shown that p120ctn-1A is related to abnormal expression of E-cadherin and poor prognosis [38]. These studies illustrated that the cytoplasmic p120ctn isoform 1A can play a role in promoting tumor cell EMT, invasion and metastasis. Based on the above, we observed on the one hand that the effect of p120ctn isoform 1A to promote tumor cell EMT, invasion and metastasis, would be lifted by its ablation, resulting in increased E-cadherin expression, decreased N-cadherin, vimentin and snail expression and inhibited invasiveness in SPC cells. On the other hand, transfection of the p120ctn isoform 1A plasmid into LK2 cells expressing cytoplasmic E-cadherin resulted in decreased E-cadherin expression, increased N-cadherin, vimentin and snail expression and enhanced cell invasiveness. Although the precise role of p120ctn during EMT induction is still unclarified, previous studies suggested that knockdown of all isoforms of p120ctn could induce EMT indirectly [27], [28], [43], [44]. All inductions were based on decreased E-cadherin expression and intercellular adhesion in previous studies, which were also confirmed by our study in H460 cells with E-cadherin membrane localization. Unlike the H460 cells, knockdown of the p120ctn isoform 1A in SPC cells with E-cadherin cytoplasmic expression could not decrease E-cadherin expression and intercellular adhesion. Instead, we found increased E-cadherin expression and decreased cell invasiveness, indicating that the EMT could not be induced by this pathway in SPC cells.

It was worth noting that the same result was observed in LK2 and H1299 cells transfected with the p120ctn isoform 3A plasmid, both showing increased E-cadherin expression, decreased N-cadherin, vimentin and snail expression and inhibited cell invasiveness. These results suggested that p120ctn isoform 3A has the function of inhibiting EMT of lung cancer cells, and this function is independent of the cellular E-cadherin localization. Past research had also confirmed a shift from p120ctn isoform 3A to p120ctn isoform 1A expression after the induction of EMT [9], [10], [11], which indirectly indicates that p120ctn isoform 3A may inhibit EMT while p120ctn isoform 1A promotes EMT. In addition, we also noticed that p120ctn and E-cadherin protein expression levels were significantly increased after transfection of the p120ctn-3A plasmid into LK2 and H1299 cells, but p120ctn and E-cadherin were still mainly restricted to the cell membrane at cell-cell adherens junctions in H1299 cells. By contrast, E-cadherin and p120ctn were almost exclusively located in the cytoplasm in LK2 cells (Figure 4E). As a cell adhesion molecule, E-cadherin is known to be only located on the cell membrane with the potential to inhibit EMT, while in the cytoplasm, it is often cleaved into fragments and therefore functions differently from the molecules located on the cell membrane [34]. Thus, the cytoplasmic E-cadherin would theoretically not play a role in inhibiting EMT. Based on the above analysis, we speculated that there may be some interaction between p120ctn isoform 3A and snail which plays a role in suppressing EMT in lung cancer cells expressing cytoplasmic E-cadherin, but this hypothesis requires further study.

Importantly, we also found that knockdown of p120ctn-1A in SPC cells with cytoplasmic E-cadherin resulted in decreased twist expression (Figure 3B). Meanwhile, transfection of LK2 cells, which also showed cytoplasmic localization of E-cadherin, with the p120ctn isoform 1A plasmid resulted in increased twist expression (Figure 4B). However, no changes in twist expression were observed in the rest of the experiments (Figure 3A, 4A). As a transcription factor and master gene regulator of EMT [39], [40], twist can downregulate E-cadherin expression [41] and upregulate N-cadherin and other mesenchymal biomarkers [42]. Increased twist expression in LK2 cells transfected with the p120ctn isoform 1A plasmid indicated that transcriptional activation took place and further suggested that the p120ctn isoform 1A may have translocated into the nucleus upon binding of E-cad/CTF2 in the cytoplasm, consequently activating the Wnt signaling pathway to promote EMT. Decreased twist expression in SPC cells transfected with p120ctn-1A-siRNA indicated that transcriptional activity was downregulated and suggested that ablation of p120ctn isoform 1A resulted in the inhibtion of EMT by removing the stimulatory effect of the Wnt signaling activity by p120ctn isoform 1A. In the H460 and H1299 cells with E-cadherin localized in the membrane, the unchanged twist expression confirmed that p120ctn isoforms 1A and 3A could bind to E-cadherin and maintain effective cell-cell adhesion in order to suppress EMT instead of affecting the Wnt/twist pathway. Intriguingly, overexpression of p120ctn isoform 3A did not change twist expression in LK2 cells expressing cytoplasmic E-cadherin, indicating that p120ctn isoform 3A did not activate transcription. Therefore, we firmly believe in the above hypothesis that p120ctn isoform 3A may interact with snail in some manner to influence E-cadherin expression and suppress EMT in lung cancer cells carrying cytoplasmic E-cadherin.

Previous studies have observed that p120ctn-1A restored the cytoplasmic expression of E-cadherin, whereas p120ctn-3A could not [20], which seems to be contradictory with the results of this study. However, the method in previous studies of knocking down p120ctn expression and then transfecting p120ctn isoforms 1A and 3A plasmids into cells is different from that in the current study in which cells were only transiently transfected with p120ctn isoforms 1A and 3A plasmids. Therefore, the different research methods may have led to different effects on E-cadherin. We also noted that in previous studies decreased and almost undetectable levels of E-cadherin by ablation of p120ctn resulted in the failure of exogenous p120ctn-1A to translocate into the nucleus to activate the Wnt/b-catenin pathway and decrease E-cadherin expression due to the deletion of the binding partner E-cad/CTF2. However, the LK2 and H1299 cell lines used in these experiments expressed E-cadherin in the present study. E-cadherin binds primarily to unphosphorylated p120ctn isoform 3A, whereas tyrosine-phosphorylated p120ctn isoform 1A interacts exclusively with N-cadherin [23]. In the previous studies, exogenous p120ctn isoform 3A was prevented from binding and stabilizing E-cadherin after its ablation, while in the present study the exogenous p120ctn isoform 3A could stabilize E-cadherin expression directly on the membrane or indirectly by increasing its cytoplasmic expression via regulation of snail expression. Of course, all of these findings will need to be further investigated.

In conclusion, we, for the first time, found that p120ctn isoforms 1A and 3A to have different functions in EMT of lung cancer cells with E-cadherin expressed in different subcellular locations. When E-cadherin was localized on the cell membrane, p120ctn isoforms 1A and 3A both could inhibit EMT and reduce the cell invasiveness phenotype. When E-cadherin was localized in the cytoplasm, p120ctn isoform 1A promoted EMT and enhanced cell invasion, while p120ctn isoform 3A inhibited EMT and reduced cell invasiveness.
